# Effectiveness and Safety of RHA4 Versus Comparator Product for Midface Volume Deficiency: A Randomized, Prospective, Multicenter Clinical Study

**DOI:** 10.1111/jocd.71113

**Published:** 2026-08-17

**Authors:** Ava Shamban, Sabrina Fabi, John Joseph, Sue Ellen Cox, Susan Weinkle, Joely Kaufman‐Janette, Jeffrey Dover, Steven Dayan, Laura Picault, Alice Melotti, Stefania Ballarini

**Affiliations:** ^1^ AVA MD Santa Monica Santa Monica California USA; ^2^ Cosmetic Laser Dermatology San Diego California USA; ^3^ The Clinical Testing Center of Beverly Hills Encino California USA; ^4^ Aesthetic Solutions Chapel Hill North Carolina USA; ^5^ University of South Florida Bradenton Florida USA; ^6^ Skin Research Institute LLC Coral Gables Florida USA; ^7^ SkinCare Physicians Chestnut Hill Massachusetts USA; ^8^ DeNova Research Chicago Illinois USA; ^9^ TEOXANE SA Geneva Switzerland

**Keywords:** aesthetic medicine, cheek augmentation, dermal fillers, hyaluronic acid, injectables, midface volume correction, multilayering technique

## Abstract

**Background:**

Hyaluronic acid (HA) dermal fillers are widely used to correct age‐related midface volume deficits and improve self‐perceived attractiveness. TEOSYAL RHA 4 (also FDA‐approved as RHA Dynamic Volume) (RHA4) is a high‐strength, moderate‐stretch HA volumizing injectable designed to maintain facial dynamics.

**Objectives:**

To evaluate the effectiveness and safety of RHA4 versus an active comparator (Juvéderm Voluma XC) for correction of midface volume deficits (MVD).

**Methods:**

In this controlled, randomized (3:1), blinded live‐evaluator, multicenter prospective clinical study, the primary outcome was the change from baseline in Teoxane Midface Volume Deficit Scale (TMVDS) score, while the co‐primary endpoint was the proportion of responders with ≥ 1‐grade improvement on the TMVDS at 8 weeks post‐treatment.

**Results:**

Overall, 201 subjects were enrolled (152 RHA4; 49 comparator); 74.6% received multilayer injections in deep and superficial midface fat pads. Mean TMVDS change at 8 weeks was −1.3 ± 0.8 with RHA4 and −1.3 ± 0.6 with comparator (*p* = 0.377; upper 95% CI bound < 0.5, non‐inferiority demonstrated). The proportion of subjects achieving ≥ 1‐grade improvement (responder rate) was 87.0% (RHA4) and 90.5% (comparator) (*p* = 0.786); the co‐primary endpoint was met (comparator responder rate > 70%). TMVDS responder rate following RHA4 treatment remained above 65.6% for 52 weeks, while subject‐assessed aesthetic improvement and satisfaction remained above 81.3% and 82.8%, respectively. RHA4 exhibited a good safety profile similar to the comparator.

**Conclusions:**

RHA4 was shown to be as effective as the active comparator for correction of MVD, providing sustained aesthetic outcomes, high satisfaction, and good tolerability.

## 1 Introduction

The midface is a central determinant of facial youth, with malar projection and full cheeks linked to attractiveness [1, 2]. Aging affects all soft‐tissue layers and leads to midface volume loss, skin laxity, and wrinkles [1, 2, 3, 4]. Similar changes occur after major weight loss, where deflation of supportive tissues produces pronounced midface hollowing [5, 6].

Cellular aging is associated with a decline in extracellular matrix (ECM) production, including reduced synthesis of hyaluronic acid (HA) and other key structural components, contributing to loss of tissue hydration and structural integrity. Additionally, aging leads to progressive bone resorption, reduced fat cell turnover, and loss of soft tissue support—key factors underlying midface volume deficiency and the primary target of volumizing interventions such as RHA4 [7]. Age‐related changes in facial appearance, including midface volume loss, have been associated with diminished self‐perceived attractiveness and reduced psychological well‐being, underscoring the relevance of effective volumization strategies for overall quality of life [8]. The growing demand for non‐surgical, minimally invasive facial rejuvenation procedures reflects a broader trend toward preventive and maintenance‐focused aesthetic medicine, driven by patient preference for treatments with minimal downtime and predictable outcomes [9]. Minimally invasive rejuvenation procedures have become increasingly popular, and hyaluronic acid (HA) dermal gels are widely used because they are quick to perform, have favorable safety profiles, and yield high patient satisfaction [9, 10]. Cheek augmentation with HA gels can also improve self‐perceived attractiveness [8, 10].

The rheological properties of HA gels depend on HA concentration, molecular weight, and degree of crosslinking [11]. Several products are FDA‐approved for cheek volumization, including Juvéderm Voluma XC, Restylane Lyft, and Restylane Contour [12, 13].

TEOSYAL Resilient Hyaluronic Acid (RHA; TEOXANE SA, Geneva, Switzerland) gels were designed with a crosslinking technology that preserves a network of entangled HA fibers and requires fewer 1,4‐butanediol diglycidyl ether (BDDE) covalent bonds for stabilization [14, 15, 16, 17]. This technology, named PNT (Preserved Network Technology), results in dynamic products that adapt to facial movements and maintain a natural appearance [14, 15, 16]. TEOSYAL RHA 4 and RHA 4 Dynamic Volume (RHA4) are a 23 mg/mL cross‐linked HA‐based injectable containing 0.3% wt/wt lidocaine or mepivacaine [18]. RHA4 is a viscoelastic, homogeneous, and biodegradable gel implant. In the United States, it has been approved for injection into the deep dermis to superficial subcutaneous tissue for correction of moderate to severe dynamic facial wrinkles and folds, such as nasolabial folds [18]. RHA4's balance of high strength and moderate stretch makes it a suitable gel for cheek augmentation and restoring volume in the midface [14, 15, 16, 17, 18, 19]. Thanks to these rheological characteristics, RHA4 may be employed in versatile injection strategies, including the use of the multilayering technique (MLT) approach targeting both the superficial and deep fat pads in the cheeks, providing structure to the cheeks while preserving facial motion in this highly dynamic area. RHA4 is now approved in the United States for injection into the subcutaneous to supraperiosteal layers for cheek augmentation and/or correction of age‐related midface contour deficiencies in patients ≥ 22 years of age [20].

This prospective, randomized, clinical study was conducted to evaluate the safety and effectiveness of RHA4 for cheek augmentation and correction of age‐related midface contour deficiencies in the United States, compared with another HA‐based product approved for the same indication (Juvéderm Voluma XC Allergan Holdings France SAS, Courbevoie, France).

## 2 Materials and Methods

### 2.1 Study Design and Population

This randomized (3:1), evaluator‐blinded, multicenter prospective study (NCT05133739) compared the safety and effectiveness of RHA4 with an approved HA comparator product for treating midface volume deficiency. The trial was conducted in eight US centers between October 2021 and January 2024.

Eligible subjects were ≥ 22 years old with Grade 2 or 3 midface volume on the Teoxane Midface Volume Deficit Scale (TMVDS) [21]. The blinded live evaluator (BLE) and primary investigator (PI) had to agree that this criterion was met; however, concordance of fullness was not required, meaning that a difference of one point between assessments was considered acceptable, and the subject was eligible for the study. Additionally, cheeks had to present the same TMVDS grade on the left and right side of the face (i.e., approximate bilateral symmetry). Subjects had to be able to follow study instructions, complete all required visits, and give their informed consent before participation.

Key exclusion criteria included hypersensitivity to lidocaine and/or amide local anesthetic agents, or any component of the devices, or a history of severe allergy or anaphylactic shock, or prior and/or planned therapies (including procedures) that would affect subject safety, or the scientific integrity of the investigation.

All injected subjects formed the safety population. The intent‐to‐treat (ITT) population included all randomized subjects. The per protocol (PP) population included ITT subjects without major protocol deviations and was used for the primary endpoint.

Ethics approval was obtained from the Institutional Review Board, Advarra, and the FDA before each center's initiation. The PI of each participating site ensured that the study was conducted in compliance with the Declaration of Helsinki and national regulations applicable to Good Clinical Practice. Informed consent adhered to specific country regulatory requirements. Image rights were obtained via informed consent.

### 2.2 Study Devices and Treatment Protocol

RHA4 contains 23 mg/mL crosslinked HA, while the comparator contains 20 mg/mL crosslinked HA. Both are BDDE‐crosslinked and contain lidocaine hydrochloride (0.3% w/w). Subjects were randomized 3:1 to RHA4 or comparator. This ratio was adopted in compliance with FDA requirements for premarket approval (PMA) clinical trials, which mandate collection of sufficient data on the investigational device to demonstrate its safety, performance, and non‐inferiority toward an FDA‐approved comparator. The BLE remained blinded, while subjects and PIs were not.

**FIGURE 1:**
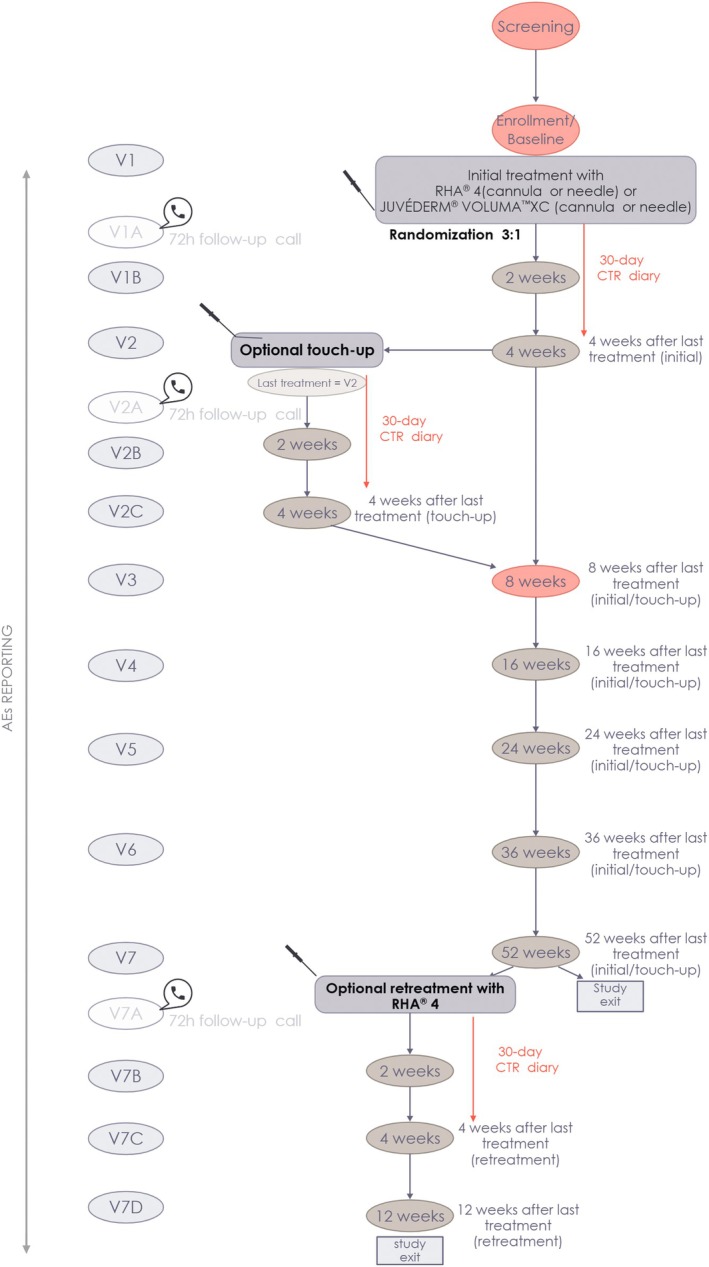
Study design. All subjects received midface treatment at Visit 1 (V1). An optional touch‐up could be performed four weeks later at Visit 2 (V2), if deemed necessary. In case of touch‐up, the subject attended additional visits at 2 weeks (V2B) and 4 weeks (V2C) following touch‐up injection. All subjects were asked to attend assessment visits at 8 (V3), 16 (V4), 24 (V5), 36 (V6), and 52 (V7) weeks after their last treatment (initial or touch‐up). Subjects either exited the study 52 weeks after the last treatment, or they were offered retreatment with RHA4 if deemed appropriate by the PI, regardless of their initial treatment (RHA4 or comparator). If the retreatment was administered, the subject attended additional visits at 2 (V7B), 4 (V7C), and 12 (V7D) weeks following retreatment.

The study design is illustrated in Figure 1.

**FIGURE 2:**
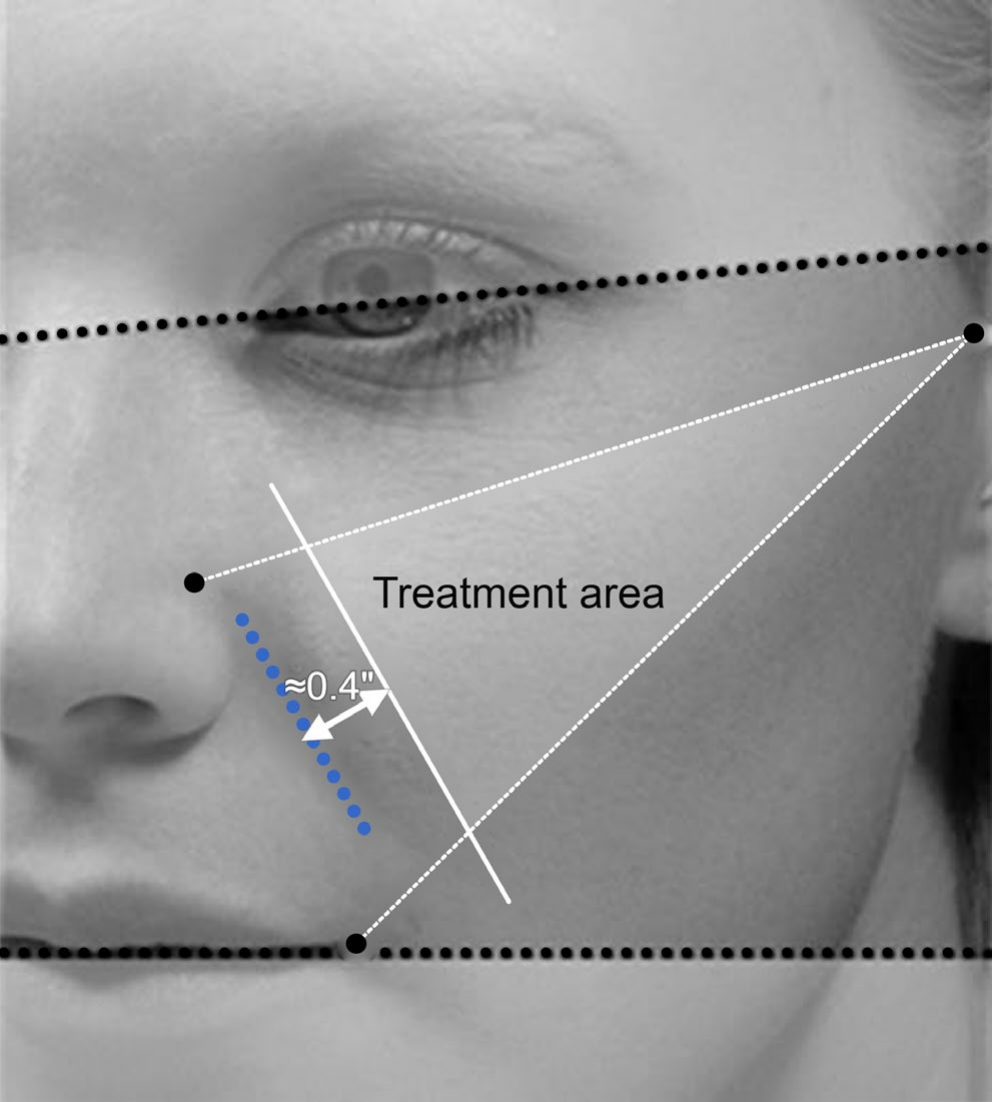
The midface treatment area. The treatment area was delimited by a triangle with a line parallel to the nasolabial fold (at least 1 cm lateral to the fold), a line from the superior part of the nasal alar crease to the superior root of the helix of the ear, and a line from the lateral oral commissure to the superior root of the helix of the ear. Treatment of nasolabial folds, temples, pre‐auricular, and periorbital regions was prohibited.

Each HA‐based product was injected into the subcutaneous and/or supraperiosteal layers of the midface treatment area at PI discretion according to evidence‐based injection guidelines, the subject's needs, and skin thickness (Figure 2) [2]. For RHA4, injections could be performed with a 27G ½ in. needle or 25G 2‐in. cannula. For the comparator product, injections could be performed with a 27G ½ in. needle, 25G 1 in. needle, or 25G 1½ in. cannula. The injection technique could include multilayering in both the subcutaneous and supraperiosteal layers with multiple‐bolus technique with needle or cannula, or fanning technique with cannula. A maximum of 6 mL of product could be administered per treatment session (3 mL per side), according to the study protocol and product instructions for use (e.g., in European countries for RHA4) [17].

### 2.3 Efficacy and Safety Assessments

#### 2.3.1 Teoxane Midface Volume Deficit Scale

Midface volume was assessed using the five‐grade photonumeric TMVDS, scored from 0 (absent) to 4 (very severe) [21]. The scale was developed and validated through a process like those described in the literature for other aesthetic scales, at the request of the FDA [3, 22, 23, 24, 25, 26]. A clinical significance exercise was also conducted on photographs to verify the scale's ability to detect a clinically meaningful difference of 1 grade between subjects or before/after treatment. The scale was validated photographically and through live subject evaluation. The scale validation protocol complied with ethical principles of the 1975 Declaration of Helsinki, and informed consent was obtained from each subject participating in this validation process. TMVDS score was assessed by the BLE at baseline (V1) and each visit from week 8 after last injection (V3) onwards (except 7B), by the PI at baseline (V1) and each visit from touch‐up visit (V2) onwards (except 2B and 7B), and by the independent photographic reviewer (IPR) at baseline (V1) and week 8 after last injection (V3). The proportion of responders with ≥ 1‐grade improvement on the TMVDS was also calculated.

#### 2.3.2 Global Aesthetic Improvement Scale

The Global Aesthetic Improvement Scale (GAIS) was used to evaluate aesthetic improvement compared with baseline, scoring appearance on a five‐grade scale from “much improved” to “much worse.” GAIS was assessed by the BLE, PI, and subject at every visit from week 4 after initial injection (V2) onwards, except V2B and V7B. The proportion of responders having a grade of “improved” or “much improved” was also calculated.

#### 2.3.3 Satisfaction

FACE‐Q^1^ Scores were used to assess satisfaction with cheeks. This was assessed by the subject with their face at rest and when smiling at baseline (V1) and each visit from touch‐up visit (V2) onwards, except V2B and V7B.

Subject satisfaction was also measured on a 5‐grade scale from “very satisfied” to “very dissatisfied” at each visit from touch‐up visit (V2) onwards, except V2B and V7B.

#### 2.3.4 Treatment Volume for Optimal Cosmetic Result

The volume required to reach optimal cosmetic result was the total volume used for initial and touch‐up injections of the population included in the safety analysis.

#### 2.3.5 Nasolabial Folds Wrinkle Severity Scale

The five‐grade Nasolabial Folds Wrinkle Severity Scale (NLF‐WSRS) was used to assess the severity of nasolabial folds (NLFs) and wrinkles [27]. It was assessed by the BLE at baseline (V1) and at each time point between 8 weeks (V3) and 52 weeks (V7) after the last injection. The scale used was developed using the same process described above for the TMVDS. In addition, it was validated both photographically and in live subjects.

#### 2.3.6 Teoxane Infraorbital Hollow Scale

The five‐grade Teoxane Infraorbital Hollow Scale (TIOHS) was used to assess the severity of infraorbital hollows [23]. It was assessed by the BLE at baseline (V1) and every visit from week 8 after the last injection (V3) onwards, except V7B. The scale used was developed using the same process described above for the NLF‐WSRS and TMVDS. In addition, it was validated both photographically and in live subjects.

#### 2.3.7 Teoxane Midface Naturalness Subject Questionnaire

The natural look of the cheeks was assessed using the Teoxane Midface Naturalness Subject Questionnaire. This was completed by the subjects at each time point from the first touch‐up visit (V2) onwards, except V2B and V7B.

#### 2.3.8 Safety

Common treatment reactions (CTRs) were recorded by subjects for 30 days post‐injection, including their severity and duration. CTRs were classified as adverse events (AE) only when their severity or duration exceeded what is normally expected. If the subject completed at least 23 days of the diary (> 75%), it was processed as if it were fully completed. CTRs present on the last recorded day of the diary were automatically considered as AEs. For subjects who completed < 22 days of the diary, the decision on whether CTRs on the last recorded day should be classed as AEs was left to PI judgment. Details of the relationship to the study procedure and device, severity, seriousness, and duration were also recorded.

Injection site pain was assessed using a 0–100 m Visual Analog Scale (VAS) immediately after injection and at 5, 15, and 30 min. Pain was considered an AE only if its severity or duration exceeded typical post‐treatment levels. Local anesthetic pre‐treatment was permitted.

### 2.4 Study Endpoints

The primary efficacy endpoint of the study was the mean change in TMVDS score from baseline to week 8 following the last injection (V3), as assessed by the BLE. The primary endpoint was met if RHA4 was statistically non‐inferior to the comparator.

The co‐primary efficacy endpoint was the TMVDS responder rate, defined as the proportion of responders with ≥ 1 grade improvement on the TMVDS 8 weeks after the last injection (V3). The responder rate needed to be ≥ 70% for the comparator product for the co‐primary endpoint to be met. This threshold reflects FDA guidance for non‐inferiority device trials, ensuring that the investigational product is compared against a comparator with established, clinically meaningful effectiveness rather than one with merely acceptable statistical performance.

The secondary efficacy endpoints of the study included the change from baseline in TMVDS score and the TMVDS responder rate, as assessed by the BLE, PI, or IPR. GAIS scores, satisfaction with cheeks (FACE‐Q), subject satisfaction, treatment volume for OCR, NLF‐WSRS scores, TIOHS scores, and Teoxane Midface Naturalness Subject Questionnaire scores were also assessed.

The safety endpoints of the study included injection site pain (VAS), CTRs reported in the 30 days following injection, and AEs.

Primary analyses were conducted on the PP population, secondary analyses on the ITT population, and safety analyses were conducted on the safety population. For the primary efficacy analysis and safety analyses, both RHA4 and the comparator product are considered in this paper. For secondary analyses, only the efficacy of RHA4 was considered unless it was deemed necessary to compare it to the comparator product.

### 2.5 Statistical Analysis

The minimum sample size to reach 90% power on the primary outcome measure (change in TMVDS score) was 92, based on a margin of error of 0.5 and an expected standard deviation of 0.7. A total of 200 subjects were required to detect any AE with an incidence rate of at least 0.5% in the overall population and at least 2.5% in the subpopulation of subjects with Fitzpatrick skin type IV–VI, with a power of 80%. The minimum population sample size was therefore 200. As a worst case, if 15% of this population had baseline TMVDS grade 4 or 5 and were excluded from the effectiveness evaluation, there would still be a sufficient sample size (> 92 subjects).

All statistical analyses were performed using SAS software version 9.4 (SAS Institute, Cary, USA). For the primary endpoint, the *p*‐value was derived from the Wilcoxon‐Mann–Whitney model by the treatment group. To determine non‐inferiority, a 95% 2‐sided confidence interval (CI) was computed. If the upper bound of the CI was < 0.5, then non‐inferiority was demonstrated. Responder rates were calculated for TMVDS, and responder rates were compared by subgroups using Fisher's exact test. Responder rates were also calculated for GAIS, subject satisfaction, NLF‐WSRS, and TIOHS. For FACE‐Q, data were Rasch transformed to a scale of 0–100, where 0 was the worst score and 100 was the best score. Frequencies and percentages were calculated for the Teoxane Midface Naturalness Subject Questionnaire.

## 3 Results

### 3.1 Subject Demographics

A total of 201 subjects were enrolled and included in both the safety and ITT populations, 173 (86.1%) formed the PP population. Of all participants, 169 (84.1%) completed the study. The PP population was used for the primary endpoint as an FDA requirement.

Of the 201 subjects in the safety and ITT population, 152 were included in the RHA4 group and 49 in the comparator group. Most (89.6%) subjects were female (Table 1). The mean age at inclusion was 55.5 ± 9.5 years. Most subjects were white (87.0%), Fitzpatrick skin type I‐III (71.1%).

**TABLE 1:** Demographics of the safety population.

Variable		RHA4	Comparator	Total
*N*		152	49	201
Age	Mean ± SD	55.4 ± 10.0	55.6 ± 7.9	55.5 ± 9.5
	22 to 56	81 (53.3%)	24 (49.0%)	
	Over 56	71 (46.7%)	25 (51.0%)	
Sex	Female	134 (88.2%)	46 (93.9%)	180 (89.6%)
	Male	18 (11.8%)	3 (6.1%)	21 (10.4%)
Fitzpatrick skin type	I	5 (3.3%)	0 (0.0%)	5 (2.5%)
	II	40 (26.3%)	15 (30.6%)	55 (27.4%)
	III	62 (40.8%)	21 (42.9%)	83 (41.3%)
	IV	23 (15.1%)	7 (14.3%)	30 (14.9%)
	V	14 (9.2%)	4 (8.2%)	18 (9.0%)
	VI	8 (5.3%)	2 (4.1%)	10 (5.0%)
Ethnicity	Hispanic or Latino	31 (20.4%)	9 (18.4%)	40 (19.9%)
	Not Hispanic or Latino	120 (78.9%)	39 (79.6%)	159 (79.1%)
	Not available	1 (0.7%)	1 (2.0%)	2 (1.0%)
Race	Missing	0	1	1
	American Indian or Alaska native	0 (0.0%)	1 (2.1%)	1 (0.5%)
	Asian	3 (2.0%)	0 (0.0%)	3 (1.5%)
	Black or African American	19 (12.5%)	6 (12.5%)	25 (12.5%)
	White	132 (86.8%)	42 (87.5%)	174 (87.0%)

*Note:* Percentages may not add to 100% due to rounding. For race, percentages may not add to 100% because a subject could be included in more than one category.

Abbreviations: *N*, number of subjects; SD, standard deviation.

Injection depth was subcutaneous, supraperiosteal, or both (multilayering, ML), with the ML technique (MLT) being most common at initial injection (74.6%, 150/201), touch‐up (59.9%, 82/201), and retreatment (46.2%, 49/106). At initial injection, 21.1% (32/152) of subjects in the RHA4 group received subcutaneous injections versus 2.0% (1/49) in the comparator group, while 3.9% (6/152) of subjects in the RHA4 group received supraperiosteal injections compared with 24.5% (12/49) in the comparator. These differences were not seen at touch‐up or retreatment. At initial injection, the cannula was only the most frequent injection method in the total population (49.3% [99/201]), followed by needle + cannula (37.8% [76/201 subjects]) and needle only (12.9% [26/201 subjects]). The cannula only method was more frequent in the RHA4 group compared with the comparator group (53.9% [82/152 subjects] in RHA4 versus 34.7% [17/49 subjects] in comparator), whereas needle + cannula was more frequent in the comparator group (33.6% [51/152 subjects] in RHA4 versus 51.0% [25/49 subjects] in comparator).

### 3.2 Primary Efficacy Analysis

At week 8 after the last injection (V3), in the PP population, the mean TMVDS grade as assessed by BLE decreased from baseline by −1.3 ± 0.8 points in the RHA4 group and by −1.3 ± 0.6 in the comparator group (Table 2). There was no significant difference between the groups (*p* = 0.377). The upper bound of the CI was less than 0.5; therefore, the non‐inferiority of RHA4 was demonstrated.

**TABLE 2:** Primary efficacy endpoint and post hoc analysis.

Variable		RHA4	Comparator
*N*		131	42
TMVDS change from baseline	Mean ± SD	−1.3 ± 0.8	−1.3 ± 0.7
	95% CI	[−1.5; −1.2]	[−1.5; −1.1]
	Min to Max	−3 to 1	−2 to 0
	95% CI for difference in means	[−0.22; 0.25]	
Responder rate	N (%)	114 (87.0%)	38 (90.5%)

Abbreviations: 95% CI, 95% confidence interval; *N*, number of subjects; SD, standard deviation; TMVDS, Teoxane Midface Volume Deficit Scale.

The proportion of subjects with ≥ 1‐grade improvement on the TMVDS (responder rate) in the PP population was 87.0% for the RHA4 group and 90.5% for the comparator group at 8 weeks (V3). To successfully achieve the co‐primary endpoint, the responder rate for the comparator group needed to be ≥ 70%. As more than 90% of subjects in the comparator group were responders, the co‐primary endpoint was met. Additionally, there was no significant difference between responder rates in RHA4 and comparator groups (*p* = 0.786).

Subgroup analyses showed that Fitzpatrick skin type, gender, ethnicity, injected volume, and administration method had no impact on TMVDS change from baseline or TMVDS responder rates, as assessed by the BLE, between RHA4 and the comparator. When analyzing only the RHA4 treatment group, a higher proportion of responders was found in younger subjects, with a statistically significantly higher responder rate of 97.2% in 22‐ to 56‐year‐olds compared with 75.0% in those over 56 years old (*p* = 0.0002). The mean TMVDS change from baseline following RHA4 treatment was numerically greater in subjects aged 22 to 56 years (*p* = 0.0852), consistent with the statistically significantly higher responder rate in this age group (*p* = 0.0002), with a change of −1.8 ± 0.5 in subjects ≤ 40 (responder rate: 100.0%), −1.3 ± 0.6 in 41 to 55 (responder rate: 97.0%), −1.3 ± 0.8 in 56 to 67 (responder rate: 82.8%), and −1.2 ± 1.0 in subjects ≥ 68 (responder rate: 71.4%) years of age, respectively. Additionally, mean TMVDS change from baseline in the RHA4 treatment group was numerically higher in subjects graded as severe at baseline, with a mean change of −1.5 ± 0.8 (responder rate: 91.4%) and −1.0 ± 0.6 in subjects graded as moderate at baseline (responder rate: 84.7%), although no formal statistical test was performed between severity subgroups.

### 3.3 Secondary Efficacy Analysis

#### 3.3.1 Treatment Sessions and Volume

The mean initial treatment volume was 2.9 ± 1.1 mL for RHA4 and 2.5 ± 1.0 mL for the comparator (Table 3). Mean touch‐up volumes were 1.3 ± 0.7 mL and 1.2 ± 0.7 mL, respectively, with touch‐up performed in 63.2% of RHA4 subjects and 83.7% of comparator subjects. The mean total volume required to achieve OCR was 3.7 ± 1.6 mL for RHA4 and 3.5 ± 1.4 mL for the comparator.

**TABLE 3:** Number of treatment sessions and treatment volume in the safety population.

Variable		RHA4 (*N* = 152)	Comparator (*N* = 49)
Patients receiving treatment	Initial	152 (100.0%)	49 (100.0%)
	Touch‐up	96 (63.2%)	41 (83.7%)
	Retreatment	77 (50.7%)	29 (59.2%)
Injection volume for both cheeks (mean ± SD)	Initial	2.9 ± 1.1 mL	2.5 ± 1.0 mL
	Touch‐up	1.3 ± 0.7 mL	1.2 ± 0.7 mL
	Retreatment	1.7 ± 1.0 mL	2.1 ± 0.8 mL
	To achieve OCR	3.7 ± 1.6 mL	3.5 ± 1.4 mL

Abbreviations: *N*, number of subjects; OCR, optimal cosmetic result; SD, standard deviation.

Retreatment at week 52, defined as RHA4 injection regardless of initial allocation, used 1.7 ± 1.0 mL in subjects initially randomized to RHA4 and 2.1 ± 0.8 mL in those initially randomized to the comparator. Retreatment was performed in 50.7% of RHA4 subjects and 59.2% of comparator subjects in the ITT population. Across the study, 37.5% of RHA4 subjects and 51.0% of comparator subjects received initial, touch‐up, and retreatment injections, while 23.7% of RHA4 subjects and 8.1% of comparator subjects received only the initial injection.

Administration methods, injection depth, and technique were chosen by investigators. RHA4 was most often injected with a cannula (53.9%), whereas the comparator was most often administered using a needle and cannula (51.0%). Injection depth was subcutaneous, supraperiosteal, or both, with MLT used initially in 75.0% of RHA4 subjects and 73.5% of comparator subjects. Common techniques included retrograde and anterograde fanning, multiple bolus for RHA4, and fanning with serial puncture or tunneling, and serial puncture alone for the comparator.

#### 3.3.2 TMVDS Responder Rate

**FIGURE 3:**
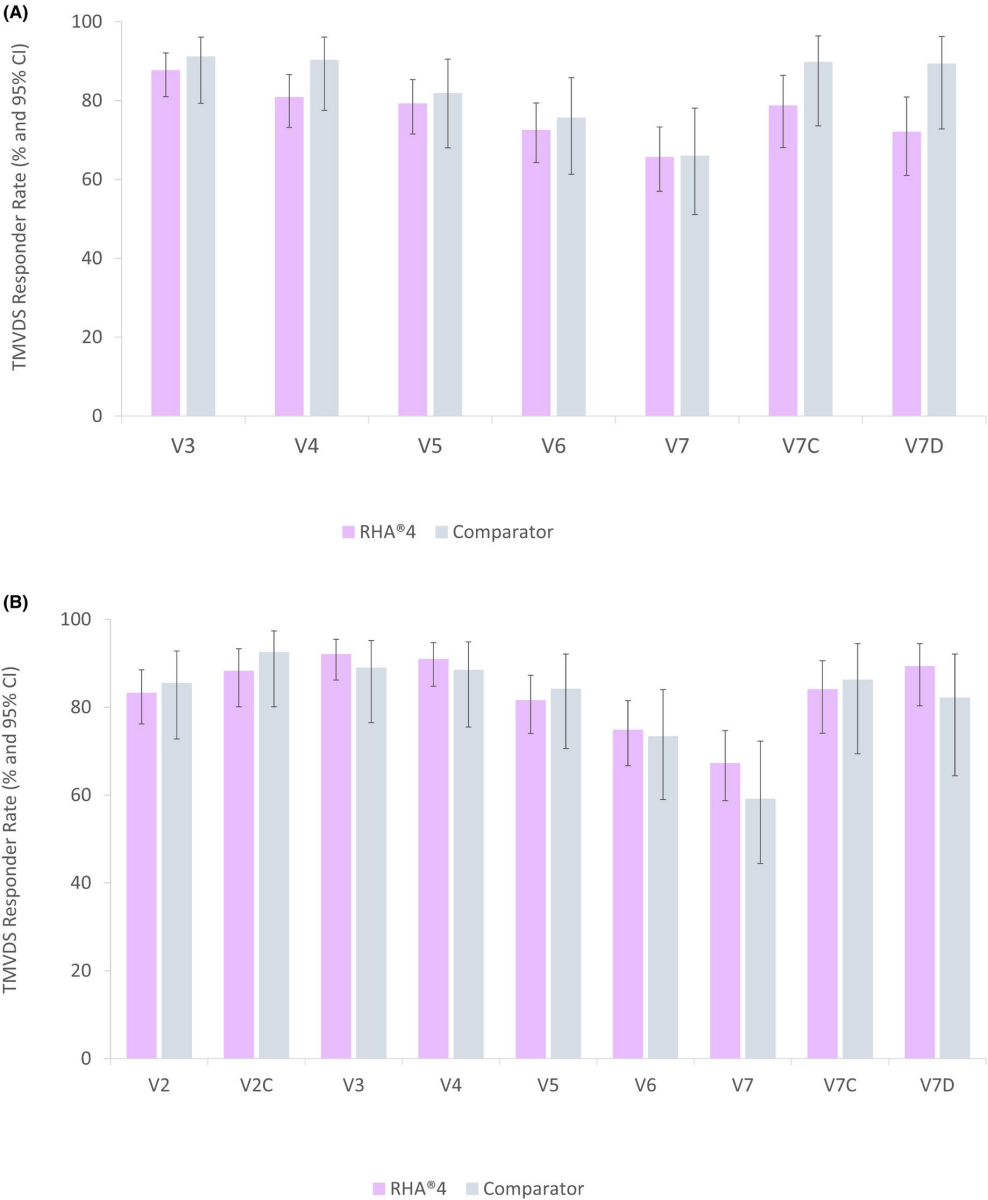
TMVDS responder rate throughout the study period. (A) Assessed by BLE. (B) Assessed by PI.

Following treatment with RHA4, the TMVDS responder rate, as assessed by the BLE, and based on the ITT population, reached 87.6% (91.1% for comparator) at 8 weeks (V3) and 79.2% (81.8% for comparator) at 24 weeks (V5) after the last treatment. It decreased by less than 25% throughout the study period, remaining at 65.6% 52 weeks following the last injection (V7) in the ITT population (Figure 3). The responder rate for the comparator group was like RHA4 at each visit, remaining at 65.9% 52 weeks post‐treatment (V7).

Similarly, the TMVDS responder rate as assessed by the PI reached 92.0% at V3 in the RHA4 group, remaining at 67.2% 52 weeks post treatment (V7). A similar response was observed across the study for the comparator product.

The TMVDS responder rate, as assessed by the PI at V3, was also similar for RHA4 (80.9%) and the comparator (82.2%). In the RHA4 group, 48.9% of subjects experienced an improvement from TMVDS grade 2 or 3 at baseline to grade 1 at V3, compared with 55.6% in the comparator group. The proportion of achieving a ≥ 2‐grade improvement was comparable between groups (38.6% in the RHA4 group and 40.0% in the comparator group). In the RHA4 group, 5.8% of subjects decreased from Grade 3 to Grade 0; no such change was observed in the comparator group. Improvement from Grade 2 to Grade 0 was observed in 7.3% of subjects in the RHA4 group and 6.7% in the comparator group.

#### 3.3.3 GAIS Improvement

**FIGURE 4:**
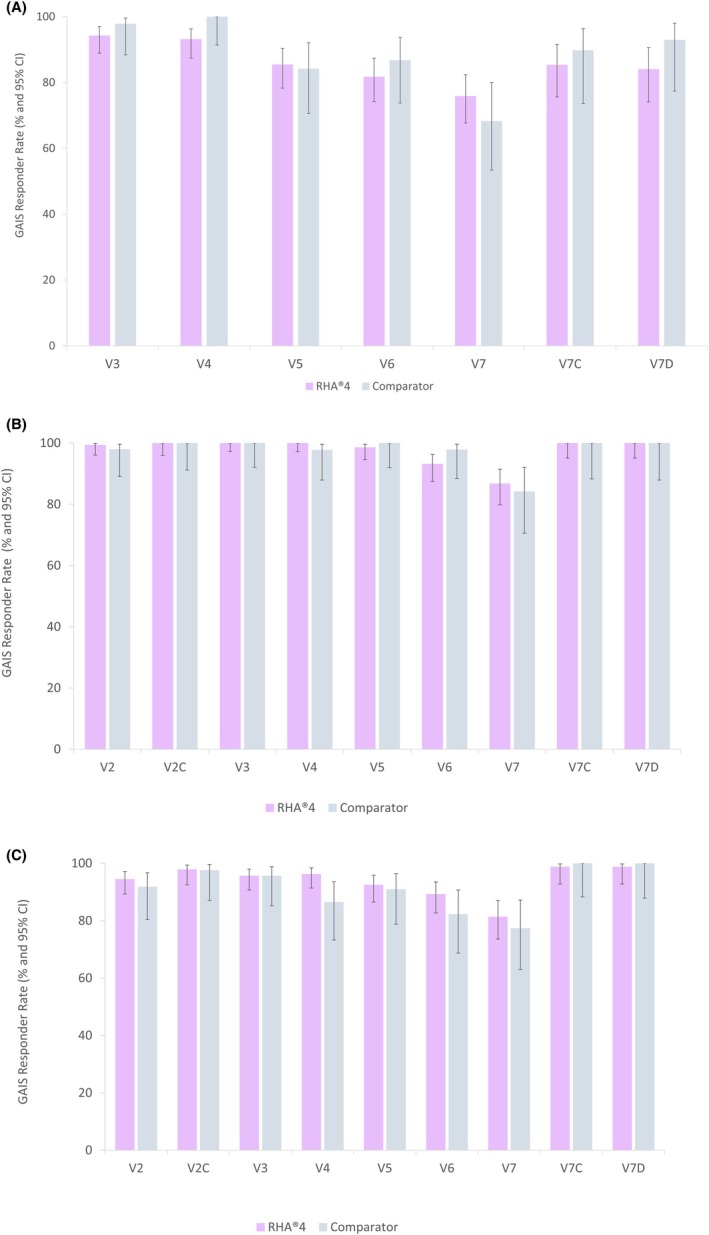
GAIS assessment throughout the study period. (A) Assessed by BLE. (B) Assessed by PI. (C) Assessed by the subject.

At 8 weeks following the last treatment (V3) with RHA4, the proportion of subjects deemed “improved” or “much improved” on the GAIS scale (responder rate) by the BLE was 94.2% (Figure 4). This remained at least 75.8% for 52 weeks of post‐treatment. The responder rates were similar for the comparator product.

The GAIS responder rate, as assessed by the PI, was 100.0% 8 weeks following the last RHA4 treatment (V3) and remained at 86.7% for 52 weeks post‐treatment (V7). When assessed by the subject, the GAIS responder rate was 95.6% 8 weeks following the last RHA4 treatment (V3), remaining at 81.3% 52 weeks post‐treatment (V7). Similar responder rates were seen for the comparator product.

#### 3.3.4 Subject Satisfaction

**FIGURE 5:**
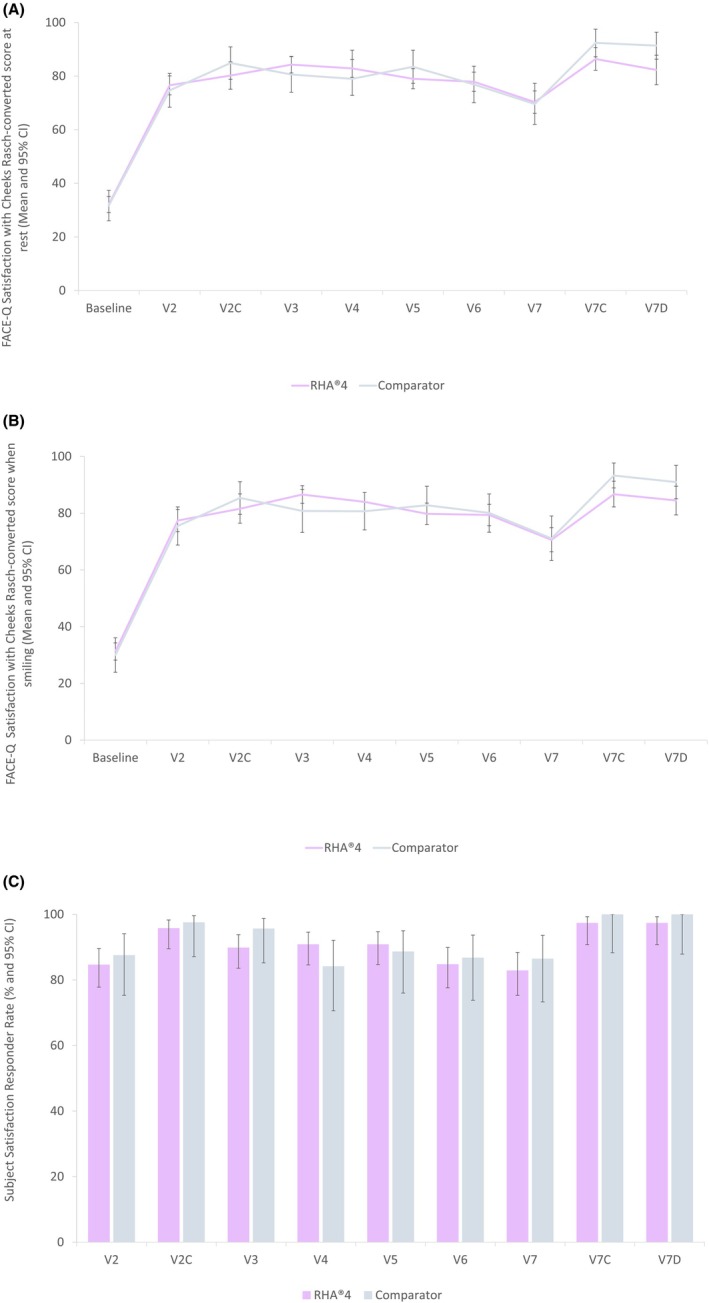
Subject satisfaction throughout the study period. (A) FACE‐Q Rasch‐converted mean score (± standard deviation) at rest. (B) FACE‐Q Rasch‐converted mean score (± standard deviation) when smiling. (C) Subject satisfaction responder rate.

In the RHA4 group, FACE‐Q satisfaction with cheeks at rest increased from 32.1 ± 18.5 at baseline to 84.3 ± 18.3 8 weeks after the last treatment (V3) (Figure 5A). Satisfaction remained high throughout the study, with a score of 70.3 ± 24.0 52 weeks post‐treatment (V7). FACE‐Q satisfaction with cheeks when smiling increased from 31.3 ± 19.1 at baseline to 86.6 ± 18.3 8 weeks after the last treatment (V3) in the RHA4 group (Figure 5B). Satisfaction when smiling remained high even 52 weeks post‐treatment (V7), with a score of 70.6 ± 24.1. Comparable results were seen with the comparator product.

When the subject satisfaction score was evaluated, 89.8% of subjects in the RHA4 group were “satisfied” or “very satisfied” (responder rate) 8 weeks after the last treatment (V3) (Figure 5C). The level of satisfaction remained high for the entire study, with a responder rate of 82.8% at 52 weeks post‐treatment (V7). The responder rates were similar for the comparator product.

#### 3.3.5 NLF‐WSRS Improvement

**FIGURE 6:**
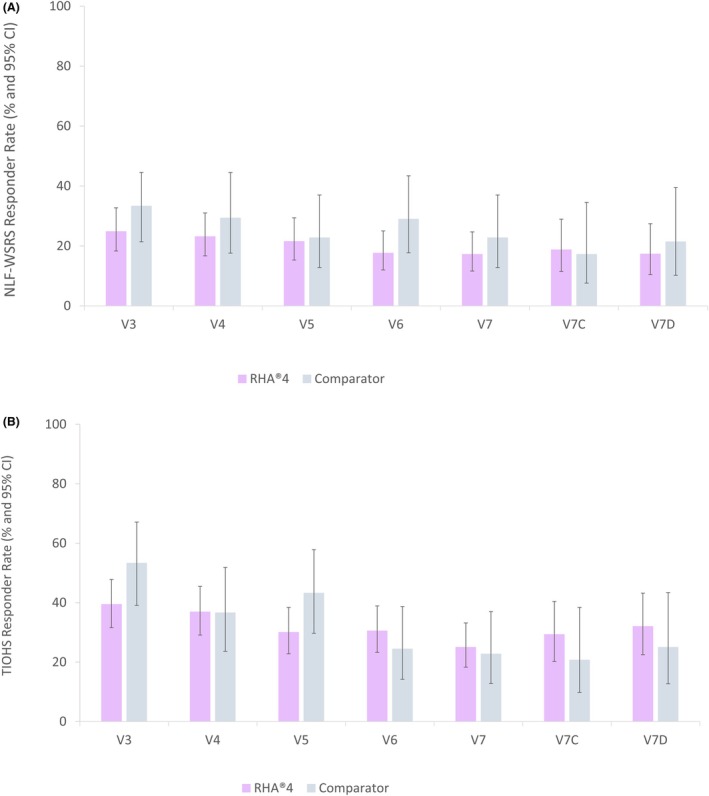
Improvement of the nasolabial folds and infraorbital hollows throughout the study period. (A) NLF‐WSRS responder rate as assessed by the BLE. (B) TIOHS responder rate as assessed by the BLE.

The severity of nasolabial folds as assessed by the BLE using the NLF‐WSRS decreased in some subjects after treatment with RHA4, with 24.8% of subjects having ≥ 1‐grade improvement on both sides (responder rate) 8 weeks after the last treatment (V3) (Figure 6A). At 52 weeks of post‐treatment (V7), the responder rate was 17.2%. The responder rates were similar for both products.

#### 3.3.6 TIOHS Improvement

The severity of infraorbital hollows as assessed by the BLE using the TIOHS decreased in some subjects in the RHA4 group, with 39.4% having ≥ 1‐grade improvement on both sides (responder rate) 8 weeks after the last treatment (V3) (Figure 6B). By 52 weeks of post‐treatment (V7), the responder rate was 25.0%. The responder rate for the comparator product was slightly higher at V3 (53.3%) but similar to V7.

#### 3.3.7 Teoxane Midface Naturalness Subject Questionnaire

In the RHA4 group, 97.8% of subjects “agreed” or “strongly agreed” that their cheeks looked natural at rest 8 weeks after the last treatment (V3). This remained high throughout the study, remaining at 95.3% 52 weeks post‐treatment (V7). Comparable results were reported for other questions of the questionnaire, including the natural look of cheeks when smiling (V3: 97.8%; V7: 95.3%) and radiance of cheeks at rest (V3: 89.0%; V7: 77.9%). Subjects also reported that they did not perceive restricted movement in cheeks when smiling (V3: 97.0%; V7: 93.8%), or when kissing (V3: 97.8%; V7: 95.4%), that they could not feel the dermal injectable when touching their cheek (V3: 91.2%; V7: 93.7%), and that their facial expressions had not changed from before treatment (V3: 93.4%; V7: 85.1%). Comparable results were seen with the comparator product.

### 3.4 Safety Analysis

Injection site pain immediately after injection with RHA4 was mostly mild to moderate at initial (7.2 ± 17.3 mm), touch‐up (8.0 ± 19.5 mm), and retreatment (8.6 ± 21.4 mm) injections. Pain drastically decreased following injection, with mean pain scores approaching zero by 15 min post‐injection. Similar pain scores were reported following injections with the comparator product at each visit.

After initial and touch‐up injections, a total of 179 AEs were reported by 87 subjects (43.3%); 136 by 68 subjects (44.7%) in the RHA4 group and 43 by 19 subjects (38.8%) in the comparator group. Of these, 84 across 44 subjects (21.9%) were considered related to the study device (adverse device effects, ADEs); 67 across 36 subjects in the RHA4 group (23.7%) and 17 across 8 subjects in the comparator group (16.3%). All ADEs across both treatment groups were mild or moderate. The most common ADEs were injection site mass or induration. There were no late‐onset or serious ADEs.

The same pattern of occurrence rates was observed after retreatment and was not influenced by Fitzpatrick type, ethnicity (Hispanic and non‐Hispanic), gender, injected volumes, or administration methods.

Following initial injection, most subjects (92.0%) reported at least one CTR; 90.8% of those in the RHA4 group and 95.7% of those in the comparator group (Table 4). The percentage of subjects experiencing a CTR was lower following touch‐up (RHA4: 79.8%; comparator: 77.5%) and retreatment (RHA4: 78.9%; comparator: 86.2%) injections. CTRs were mostly mild or moderate and lasted 1 to 3 days.

**TABLE 4:** Common treatment reactions reported by subjects in the 30 days following injection.

Injection	Reaction	RHA4	Comparator	Total
Initial	*N* subjects	152	49	201
	*N* diary entries	141	46	187
	Any CTR	128 (90.8%)	44 (95.7%)	172 (92.0%)
	Redness	61 (43.3%)	17 (37.0%)	78 (41.7%)
	Pain	70 (49.6%)	20 (43.5%)	90 (48.1%)
	Tenderness	113 (80.1%)	39 (84.8%)	152 (81.3%)
	Firmness	108 (76.6%)	34 (73.9%)	142 (75.9%)
	Swelling	98 (69.5%)	28 (60.9%)	126 (67.4%)
	Lumps/bumps	77 (54.6%)	23 (50.0%)	100 (53.5%)
	Bruising	74 (52.5%)	16 (34.8%)	90 (48.1%)
	Itching	17 (12.1%)	2 (4.3%)	19 (10.2%)
	Discoloration	32 (22.7%)	6 (13.0%)	38 (20.3%)
Touch‐up	*N* subjects	96	41	137
	*N* diary entries	94	40	134
	Any CTR	75 (79.8%)	31 (77.5%)	106 (79.1%)
	Redness	27 (28.7%)	12 (30.0%)	39 (29.1%)
	Pain	32 (34.0%)	12 (30.0%)	44 (32.8%)
	Tenderness	57 (60.6%)	28 (70.0%)	85 (63.4%)
	Firmness	50 (53.2%)	18 (45.0%)	68 (50.7%)
	Swelling	45 (47.9%)	18 (45.0%)	63 (47.0%)
	Lumps/bumps	33 (35.1%)	17 (42.5%)	50 (37.3%)
	Bruising	38 (40.4%)	14 (35.0%)	52 (38.8%)
	Itching	8 (8.5%)	2 (5.0%)	10 (7.5%)
	Discoloration	14 (14.9%)	7 (17.5%)	21 (15.7%)
Retreatment	*N* subjects	77	29	106
	*N* diary entries	76	29	105
	Any CTR	60 (78.9%)	25 (86.2%)	85 (81.0%)
	Redness	22 (28.9%)	10 (34.5%)	32 (30.5%)
	Pain	23 (30.3%)	14 (48.3%)	37 (35.2%)
	Tenderness	45 (59.2%)	22 (75.9%)	67 (63.8%)
	Firmness	34 (44.7%)	19 (65.5%)	53 (50.5%)
	Swelling	34 (44.7%)	16 (55.2%)	50 (47.6%)
	Lumps/bumps	28 (36.8%)	8 (27.6%)	36 (34.3%)
	Bruising	19 (25.0%)	12 (41.4%)	31 (29.5%)
	Itching	4 (5.3%)	3 (10.3%)	7 (6.7%)
	Discoloration	9 (11.8%)	4 (13.8%)	13 (12.4%)

Abbreviations: CTR, common treatment reaction; *N*, number of subjects or diary entries.

Administration with a cannula appeared to be associated with a numerically lower incidence of CTRs than administration with a needle or both devices, although no formal statistical comparison was performed. The comparison of ADE occurrence by administration method in RHA4 subjects confirmed the similarity in ADE occurrence; no statistical difference was observed between the 3 administration methods.

The comparison of ADE incidence based on the injection technique could not be made because of the different injection techniques used across treatment groups; additionally, the number of subjects in some subgroups was low.

Only 4 severe but classified as unrelated either to the device or the procedure SAEs were reported during the study.

## 4 Discussion

Midface volume is central to facial youth and attractiveness, with volume deficiencies commonly associated with aging [1, 2]. RHA4 is a high‐strength, moderately elastic gel designed for volumization while maintaining facial dynamics [14]. This study evaluated its performance for cheek augmentation and correction of midface contour deficiencies across a diverse US population. We have demonstrated that in the PP population, as requested for the primary endpoint by the FDA, RHA4 is statistically non‐inferior to a commercialized comparator product in subjects with moderate to severe midface volume deficiency on both sides of the face (TMVDS grades 2 to 3). In the full ITT population, RHA4 provided sustained midface volumization, with TMVDS responder rates of over 65% and GAIS responder rates of over 75%, even 52 weeks post‐treatment. Similarly, throughout the study, subject satisfaction with cheeks (FACE‐Q) remained above 70/100, subject satisfaction score remained above 80%, and nearly all subjects agreed that their cheeks looked natural. Some subjects also exhibited benefits on their NLFs and infraorbital hollows as indirect aesthetic outcomes of the midface treatment, with responder rates of 20% and 30% on the NLF‐WSRS and TIOHS, respectively, 24 weeks post‐treatment with RHA4. Similar effectiveness was observed with the comparator product using comparable volumes of product, although numerically fewer subjects in the RHA4 group required touch‐up and retreatment injections to achieve comparable results (no formal statistical comparison performed). Collectively, these results support the clinical use of RHA4 for cheek augmentation and correction of moderate‐to‐severe midface volume deficiency, with the product proving just as effective as the FDA‐approved, market‐leading commercialized comparator product. Of note, in a pivotal study, the active comparator showed a percentage of response of 85.6%, defined as achievement of at least one grade of improvement in a 6‐point Mid‐Face Volume Deficit Scale, and in a population including mild volume loss cases [3]. RHA4 was also well‐tolerated, with no serious ADEs and no late‐onset reactions reported. Concerning the administration method, while no differences were shown in terms of performance with needle and/or cannula, the cannula‐only administration of RHA4 was observed to have a numerically lower incidence of CTRs than RHA4 administration with a needle or both devices (no formal statistical comparison performed).

Considering the differential mobility and mechanical constraints of each layer of the midface, a multilayer treatment algorithm has been proposed, which is tailored to patient‐specificities, including age, gender, skin type, and morphology [2]. This treatment approach is considered appropriate to address volume deficit and age‐related changes affecting all layers of the midface [28]. Therefore, the concomitant treatment of two different subcutaneous planes is believed to be beneficial, especially in older subjects. The current study is the first to evaluate the effectiveness and safety of RHA4 when using an MLT for cheek augmentation, with the study protocol allowing for injection into the subcutaneous and/or supraperiosteal layers of the midface. Our results showed that while patients aged 56 or younger had higher TMVDS responder rates, RHA4 treatment also proved effective in older patients, supporting the effectiveness of the MLT in addressing overall tissue aging. Although injection technique and plane were not mandated by protocol beyond compliance with each product's instructions for use, both of which permit injection from the supraperiosteal to subcutaneous layers, technique selection was similar between groups in practice, with MLT used in 75.0% of RHA4 subjects and 73.5% of comparator subjects at initial injection, supporting comparability between treatment arms.

In addition to age, the primary endpoint was also assessed by other parameters such as Fitzpatrick skin type, total volume injected, and administration method. No differences were observed between RHA4 and the comparator or within the RHA4 group in any subgroup, suggesting that RHA4 treatment can provide benefit to all individuals. These findings are particularly interesting for subjects with Fitzpatrick skin types IV‐VI, as these individuals are often underrepresented in clinical trials [29].

The follow‐up period of the study was 52 weeks, allowing evaluation of the long‐term benefits of RHA4. The benefits of treatment with RHA4 remained evident in most subjects for 52 weeks of post‐treatment. These long‐lasting benefits are likely due to crosslinking technology, which allows for lower rigidity and improved adaptability to facial dynamics [14]. However, previous cheek augmentation studies have found benefits lasting up to 2 years following treatment with other HA fillers [3, 29, 30]. Given the sustained benefits of RHA4 even 52 weeks of post‐treatment, future studies should include a 2‐year follow‐up period to further assess the longevity of RHA4.

Prior research has evaluated RHA4 for the treatment of moderate‐to‐severe NLFs, confirming the efficacy and safety in this indication [16]. Our study suggests that midface treatment with RHA4 may confer indirect benefits for NLFs and infraorbital hollows, although this is not guaranteed. For subjects who need treatment in a broader area, including the NLF, RHA4 could be used first to treat the midface and second in the NLF, which is already a granted indication, thus offering a comprehensive approach to midface rejuvenation.

The choice of products should be based on their biocompatibility, tissue integration, and mechanical and rheological properties, which should match the tissues' characteristics and biomechanics. The most desirable filler properties are creating both deep structural support and facial movement. Thanks to the development of the RHA collection based on the PNT, with relatively lower content of cross‐linked HA and highly dynamic properties, injection of superficial fat pads in the midface has become possible with RHA4 [2, 28].

This study is limited by the unblinded design; however, this bias was mitigated by the assessments performed by the BLE. Furthermore, the investigators, BLEs, and IPRs underwent training in using the proposed scales, ensuring consistency in results throughout the study. Additionally, the unequal (3:1) randomization ratio between study groups, required by FDA PMA regulations, limits the direct comparability of group‐level analyses. Furthermore, the study population was predominantly female (89.6%) and of Fitzpatrick skin types (I–III, 71.1%), which may limit the generalizability of findings to more diverse patient populations, although FDA‐mandated minimum thresholds for inclusion of male subjects (≥ 10%) and Fitzpatrick types IV–VI (≥ 20%, including ≥ 10% types V–VI) were met [3, 29, 31, 32]. Another potential limitation resides in the proprietary nature of certain efficacy parameters, such as the TMVDS, NLF‐WSRS, and TIOHS, and different scales used in other studies sponsored by the manufacturers of alternative products, preventing direct comparison. Nevertheless, secondary efficacy endpoints, including widely used methods, such as GAIS, FACE‐Q scores, and subject satisfaction, can provide consistent and robust results that provide overall support for the effectiveness of RHA4 for the treatment of midface volume deficiency.

## 5 Conclusion

This study has demonstrated the effectiveness and safety of RHA4 for the correction of moderate‐to‐severe midface volume deficiency. RHA4 was at least as effective as the FDA‐approved, market‐leading comparator product, providing sustained measurable improvement in midface volume deficit severity, as well as elevated levels of global aesthetic improvement and subject satisfaction. Subjects also declared that the treatment outcomes looked natural, with some showing broader aesthetic indirect benefits in their NLFs and infraorbital hollows. While similar volumes of RHA4 and comparator were used to achieve these clinically relevant effects, numerically fewer treatment sessions were required in the RHA4 group. The study allowed for the use of a technique, if required, combining injections in the subcutaneous and supraperiosteal planes. While RHA4 was more effective in subjects aged 56 years and younger, the MLT enabled older subjects to also be treated effectively. RHA4 was well tolerated, with no late‐onset reactions or serious ADEs. No safety concerns were associated with the use of the MLT, nor with the use of a needle and/or cannula. These results supported the expansion of the RHA4 indication in patients aged 22 years or older in the United States to include cheek augmentation and correction of age‐related midface contour deficiencies, in addition to its existing nasolabial fold indication [20]. Future studies could evaluate RHA4 in combination with other products for a comprehensive approach to facial rejuvenation.

## Author Contributions

A.S., S.F., J.J., S.E.C., S.W., J.K.‐J., J.D., and S.D. were involved in data generation while performing their roles as investigators in the clinical trial. L.P., A.M., and S.B. are employees of TEOXANE SA Clinical Development and Medical Affairs. L.P. was involved in the study and data collection conduct, A.M. supervised the study and data analysis, and S.B. contributed to the medical writing of the manuscript. All authors contributed to data interpretation and final manuscript review.

## Funding

This work was supported by TEOXANE SA.

## Disclosure

The authors were clinical investigators for this study and received research grants from the study sponsor, TEOXANE, for their participation in the study.

## Ethics Statement

Ethics approval was obtained from the Institutional Review Board, Advarra, prior to each center's initiation. The primary investigator of each participating site ensured that the study was conducted in compliance with the Declaration of Helsinki, the Health Insurance Portability and Accountability Act (HIPAA), and national regulations applicable to Good Clinical Practice.

## Consent

Informed consent adhered to specific country regulatory requirements. Image rights were obtained via informed consent.

## Conflicts of Interest

The authors declare no conflicts of interest.

## Data Availability

The data that support the findings of this study are available on request from the corresponding author. The data are not publicly available due to privacy or ethical restrictions.
